# Shared decision-making in mental health care—A user perspective on decisional needs in community-based services

**DOI:** 10.3402/qhw.v11.30563

**Published:** 2016-05-09

**Authors:** Katarina Grim, David Rosenberg, Petra Svedberg, Ulla-Karin Schön

**Affiliations:** 1 School of Health and Social Work, Dalarna University, Falun, Sweden; 2 Department of Social Work, Umeå University, Umeå, Sweden; 3 School of Social and Health Sciences, Halmstad University, Halmstad, Sweden

**Keywords:** Shared decision-making, information needs, mental health care, directed content analysis

## Abstract

**Background:**

Shared decision-making (SDM) is an emergent research topic in the field of mental health care and is considered to be a central component of a recovery-oriented system. Despite the evidence suggesting the benefits of this change in the power relationship between users and practitioners, the method has not been widely implemented in clinical practice.

**Objective:**

The objective of this study was to investigate decisional and information needs among users with mental illness as a prerequisite for the development of a decision support tool aimed at supporting SDM in community-based mental health services in Sweden.

**Methods:**

Three semi-structured focus group interviews were conducted with 22 adult users with mental illness. The transcribed interviews were analyzed using a directed content analysis. This method was used to develop an in-depth understanding of the decisional process as well as to validate and conceptually extend Elwyn et al.'s model of SDM.

**Results:**

The model Elwyn et al. have created for SDM in somatic care fits well for mental health services, both in terms of process and content. However, the results also suggest an extension of the model because decisions related to mental illness are often complex and involve a number of life domains. Issues related to social context and individual recovery point to the need for a preparation phase focused on establishing cooperation and mutual understanding as well as a clear follow-up phase that allows for feedback and adjustments to the decision-making process.

**Conclusions and Implications for Practice:**

The current study contributes to a deeper understanding of decisional and information needs among users of community-based mental health services that may reduce barriers to participation in decision-making. The results also shed light on attitudinal, relationship-based, and cognitive factors that are important to consider in adapting SDM in the mental health system.

Shared decision-making (SDM) is an emerging research topic in the field of mental health care and is considered to be a central component of a recovery-oriented system (Matthias, Salyers, Rollins, & Frankel, 2012; Onken, Craig, Ridgway, Ralph, & Cook, 2007; Slade et al., 2012). This growing interest reflects a changing view of the power relationship between users and practitioners. The development of suitable strategies in order to plan psychosocial interventions and mental health treatment is an active process that involves complex decision-making between the user and the practitioner. Interventions are however often designed to increase the user's compliance with the practitioner's view of optimal treatment (Deegan & Drake, 2006). SDM builds on a very different power relationship than compliance-oriented processes because it considers both the user and the practitioner as two experts who must share their respective information and collaboratively agree upon the choice of intervention. SDM provides a model for assessing a treatment's advantages and disadvantages within the context of recovering a life following an experience of mental illness (Deegan & Drake, 2006). Contemporary, evidence-based approaches to the management of long-term somatic illnesses are based on the process of SDM (Duncan, Best, & Hagen, 2010; O'Connor et al., 2007). SDM is advocated because of its potential for improving the quality of the decision-making process for users and as a tool for contributing to the active engagement and participation of the user in their own care and illness management (Légaré et al., 2010).

Even though there is evidence that SDM improves the quality of information exchange between users and providers in somatic care (Elwyn et al., 2013), there is still a need for SDM to be adopted in social as well as psychiatric services (Deegan & Drake, 2006; Hamann, Kruse, Schmitz, Kissling, & Pajonk, 2010; Jormfeldt, Hansson, Svensson, & Svedberg, 2014; National Board of Health and Welfare, 2011). Reported effects of implementing SDM in somatic care have been a motivation for the providers and have positive impact on the clinical process and on the user outcomes (Hamann et al., 2010). The relatively extensive knowledge presented about SDM in recent years presents three basic prerequisites for successful implementation of SDM in care settings: 1) The attending staff has the ability and is willing to include the user in decisions. 2) The user is willing and has the ability to actively participate in the decisions. 3) Additional information and decision support are available to facilitate the SDM process (Loh et al., 2007; O'Connor et al., 2009, 2007). Decision support tools adapted to the needs of users have the potential to restructure how people with mental illness and staff work together to arrive at shared decisions about the next steps in support and treatment (Deegan, 2007; National Board of Health and Welfare, 2011). Decision support tools have been developed and successfully adapted in somatic care (O'Connor et al., 2007) but are still lacking within community-based mental health services (Adams, Drake, & Wolford, 2007).

Decisional and information needs not only refer to the information and knowledge user's need regarding mental health services, but also to the need to develop a process in which staff acquire necessary information and knowledge about the user's goals, experiences, and life situations. Elwyn et al. (2013) have developed a three-step model for clinical practice to promote SDM, including “choice talk, option talk, and decision talk,” which might be usefully applied in attempts to strengthen the development of decision tools in community-based mental health services.

## Aim

The objective of this study was to investigate decisional and information needs among users with mental illness as a prerequisite for the development of a decision support tool aimed at supporting user participation in SDM. A further aim was to explore whether Elwyn's three-step model is adaptable to these decisions and decision-making processes encountered in community-based mental health services.

## Methods

### Design

A qualitative descriptive design, with both an inductive and deductive approach (Hsieh & Shannon, 2005), was used to develop knowledge about users’ decisional and information needs in mental health care. Ethical approval for the study was obtained from the Regional Ethical Review Board in Umeå (Dnr: 2012-198-31).

### Participants and data collection

Participants were recruited through user panels, mental health services, and through user organizations at three different sites in Sweden. The inclusion criterion was adults with a psychiatric diagnosis and with personal experience of using mental health services. User panels, whose members comprise the majority of the focus group respondents, consist of individuals who have been trained in basic research techniques and consult with researchers on projects, offering a user perspective. These panel members, as trained users, may tend to place less emphasis on their own individual experiences and contribute as representatives for a group of people that share experience from the same or similar phenomena as themselves. The remainder of the focus group participants contributed with more personal narratives that, although less representative, may be considered to have potential for discovering various phenomena that might not emerge in more developed presentations of the wider user experience (Strøm & Fagermoen, 2014). The mix of users and perspectives allowed for both a range of experience and an opportunity to identify a broad range of opinions and needs with a limited number of participants. After being informed orally and in writing about the aim of the study, all participants volunteered to take part in the project. Participants were also informed that at any time they could withdraw their consent to participate. In total 22 adult users, 17 women and 5 men, ranging in age from 24 to 62, participated in the interviews.

Three focus group interviews were conducted, lasting from 60 to 90 min, and there were 5–9 participants in each group (Patton, 2002). One researcher was present in the first two groups and two were present in the third. A predefined thematic interview guide was constructed and piloted with other users in one of the user panels. Only one question was reformulated prior to the focus group interviews. Respondents were contacted by phone or e-mail prior to the interviews in order to confirm that they were still interested in and understood the conditions of the study with regard to participation and confidentiality. All focus group interviews were tape-recorded and transcribed verbatim. The respondents were numbered during the transcription process to increase transparency so that the respondents’ opinions were identifiable during the analysis. After introducing the participants to the concept of SDM they were asked to reflect upon experiences of involvement when decisions are made concerning psychiatric care or treatment. It was made clear that by mental health services, we meant municipal, social, and residential supports, as well as county-based outpatient psychiatry, the two primary actors comprising the mental health system in Sweden. The focus was on informational needs: the types of knowledge a user needs and what information participants consider important for a provider to receive with regard to the user's perspective. Their views on the form as well as content of the information provided were of interest.

### Data analysis

Consistent with the aim of our study, we analyzed the data using a directed content analysis (Hsieh & Shannon, 2005). This method is used to validate and conceptually extend a model or theory (Hsieh & Shannon, 2005). The data analysis consisted of both an inductive and deductive approach. The analysis began with reading all transcribed data inductively several times. The inductive approach included defining codes through highlighting words from the text that appeared to capture key thoughts or concepts. Preliminary labels for codes emerged from the text that was reflective of more than one key thought. Codes were then sorted into categories. These emergent categories were used to organize and group codes into clusters (Patton, 2002). The deductive process involved going back to the data and placing the inductively derived codes into categories based on the three domains described by Elwyn et al. (2013). This was done in order to explore its potential for structuring our data and for its transferability to mental health services. A categorization matrix (Hsieh & Shannon, 2005) was developed from Elwyn's model in which all the data were reviewed for content and coded for correspondence with these three domains. Codes that did not fit the categorization frame were used to generate new categories with the aim of broadening the understanding of SDM in mental health services. To enhance the quality and validity of the analysis, two researchers conducted the coding independently and the data analysis was discussed continuously with the other members of the research team.

## Results

The findings from this study show a high level of agreement with the model presented by Elwyn et al. (2013). The process of SDM, as well as the content of the three steps in the model, recurs in what these users of mental health services described. However, some elements that emerged in the analysis are not readily encompassed within the three-step model. These results suggest two additional steps to Elwyn's model. First an initial step, *Preparation* concerning preparatory issues for provider tasks as well as for user tasks, and a fifth, *Follow-up* concerning needs and wishes for further contacts with the provider after a decision has been made. Elwyn's model with the extensions that emerged in this study is illustrated in Table I.

**Table I:** A model for shared decision-making in mental health services

*1. Preparation*	2. Choice talk	3. Option talk	4. Decision talk	*5. Follow up*
***Develop agenda*** ***Provide user decision suppor***	Step back Offer choice Justify choice—preferences matter Check reaction Defer closure	Check knowledge List options Describe options—explore preferences Harms and benefits Summarize	Focus on preferences Elicit preferences Move to a decision Offer Review	***Accessible contact*** ***Planned follow-up*** ***Possibility to reconsider***

**D E C I S I O N S U P P O R T** 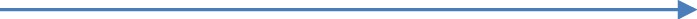				

In addition, it was noted that certain aspects of the model might need to be emphasized with respect to some of the characteristics of need expressed by the respondents. There is a pronounced focus in Elwyn's model on the communicative skills of providers. However, the respondents of this study described how they frequently feel regarded as untrustworthy, as a consequence of being mentally ill. Their focus, when referring to provider–user interaction often concerns relational aspects that go beyond that of accuracy in exchanging information. Regardless of the topic discussed, the ability of the provider to create a dialogue characterized by trust, genuine interest, respect, and equality is described as essential. Distrust in a user's sense of reality may lead the provider to dismiss or reject their input. References were made to their experiences of being exposed, feeling inferior, and being dependent in the role of a mental health service user.

but it′s just that … that′s my greatest experience of psychiatry, you aren′t taken seriously … no … others defined my reality the whole time … and certain realities weren′t allowed … (22)

Elwyn et al. (2013) describe deliberation as a key concept in the SDM process, emphasizing the importance of supporting the user to consider information about the pros and cons of their options and to consider the practical as well as emotional implications of their decision. Deliberation encompasses the need to work collaboratively with professionals as well as with members of their network that the user may prefer to turn to. The respondents in our study frequently referred to the potential benefits of involving others in the process and at a time or place of their own choice; a finding that coincides with Elwyn's suggestion that allowing room for deliberation with other people in the wider network of the user is an important aspect of SDM. However, the respondents identified some additional reasons for involving persons from their network, in addition to deliberation before and between encounters. Involvement of others was to a large extent considered a support *during* provider encounters. The advantages of being accompanied by someone who knows you well for the purpose of speaking on your behalf, as well as helping to remember and understand what is discussed in the meeting, were pointed out by many. Family members and partners or friends were suggested, in addition to professional or peer supports, as being helpful in ensuring that the user was able to convey their situation and needs in the way that they wanted to. Some noted the ways in which the mere presence of such a person might have a counterbalancing impact on the power imbalance experienced in the meeting.

### Preparation

While the Elwyn et al. (2013) model begins when the problem has already been defined, the results from this study suggest that information needs to be shared at an earlier stage than the original model proposes in order to give the user a chance to consider the need for and nature of the decision to be made, thereby extending the scope of the opportunity for deliberation. Contributions to the proposed preparation phase, which might serve to promote user involvement from the very onset, are suggested for both the provider and user. The respondents expressed a wish to receive information prior to meeting a provider, regarding the purpose and estimated duration of the meeting. This would enable the user to come prepared and would help the user in pinpointing what issues he or she wants to add to the agenda. Respondents describe how they, prior to meetings, make lists of issues they want to have addressed and questions to be answered. Lists not only serve as a memory support but are also noted by some to be an effective way to clarify for themselves and communicate to the provider the issues which they consider to be the most important to address. Some participants noted that a worksheet could serve as a support when preparing for a meeting. They expressed that receiving some kind of support tool for preparation would also serve as an expression of interest on the part of the provider.

In the descriptions of what information participants wish the provider to be aware of prior to the meeting, information concerning issues related to their experiences of their illness and various interventions would be considered valuable. This points to the importance of having mutual access to information and seems to reflect how mental health–related decisions entail certain challenges which go beyond those of health-care decisions that are more narrowly focused on medical issues. The respondents discussed the diversity of life situations that affect and are affected by their experience of dealing with mental illness. There was a belief among the respondents that they as users possess experimental knowledge, which is crucial for the decision-making process and which must be a factor that contributes to the frame for the decision to be discussed, even prior to the onset of the actual decision-making process.

So it is probably very important that I also think about what I want to share with you? What can be important for this person (the provider) to know in order to help me in the best way? (9)

Being offered the opportunity to prepare for the meeting is described as an indicator of mutuality, a factor that many respondents described as a prerequisite for a participatory decision-making process. They described the importance of concrete aids, for considering and contributing to the preparation of the decision-making occasion, that might reduce power differentials, which could act as obstacles to their participation.

A good way to communicate may be via the computer … because sometimes you must protect yourself … you are very vulnerable … Or an ordinary letter. Perhaps you can read it together with someone. (18)

The use of support tools is included in Elwyn's model as they might aid users in exploring options and clarifying preferences. In the present study, support for user decision tools introduced early in the process is clear and considered as essential to a concrete expression by the provider of inviting the user to the process as an active participant.

### Choice talk

To justify the notion of choice, Elwyn's model describes the provider's role as that of clarifying how users may generate decisions more in keeping with their personal priorities and preferences. The idea of personal choice was indeed expressed to be of great value by the respondents, but the need for clear guidance was also presented as an essential factor for feeling confident in the decision-making process.

Respondents were aware that uncertainties on the individual level regarding the effects of a particular intervention may make straightforward guidance difficult. Factors described as affecting mental health conditions are numerous, diverse, and interactive in a way which makes causes and effects in changes often difficult to pinpoint. Furthermore, some expressed how effects of treatment and support interventions of various kinds are often delayed. In light of this insecurity, participants expressed a desire to allow for a process of jointly exploring the manner in which different possibilities might lead to different results over time.

… and then you can always try … and you can always stop … (important) to feel that you are a free person. (20)

Elwyn et al. (2013) describe their model as a “process of moving from initial to informed preference” and stresses how the task of providing high-quality evidence-based information must be taken on by providers from the start to ensure that options are not considered by a person who is insufficiently informed. The respondents reported that they themselves seek information from various sources and some expressed a wish for providers to offer guidance in how to seek relevant knowledge. The Internet was described as an invaluable resource for finding general information and pertinent research, in addition to experience-based knowledge. However, preferences differed among the respondents regarding channels and formats for information and communication. These preferences and needs were described as relating to cognitive difficulties or emotional sensitivities associated with their mental illness. Verbal information was essential, often in combination with written information. Other suggestions were films and audio recordings. Similar to Elwyn et al. (2013), the participants requested individualized comprehensive information about health status and functional ability.

After verifying that the user has accepted the idea of being involved in a decision-making process, the final task within choice talk entails expressing the possibility of deferring closure. The respondents in this study similarly expressed the importance of not feeling pressured into decisions that they are not ready for.

### Option talk

In option talk, detailed information is provided regarding treatment options (Elwyn et al., 2013). The first task entails eliciting what the user already knows and whether it is accurate. The respondents similarly gave voice to the need for users to be open to having their knowledge checked and possibly corrected. They noted the need for the user to trust the knowledge of a provider because the information acquired as “a layman” from various sources may be partial, irrelevant, or contain errors. At the same time, users state that they want professionals to take into consideration not only their experiences but their general knowledge as well. Some noted how they have appreciated occasions when providers have expressed an interest in questions regarding information they acquired and might be ready to look up and into things with which they are not familiar. Comments made in reference to this imply that such action not only contributes to creating a common ground but also enhances the user's experience of reciprocity and of respect for his or her competence.

Next, Elwyn's model suggests that the provider develops a clear list of possible alternatives in order to establish a dialog in which the preferences of the user are explored along with the various benefits and risks they each may entail. In the current data, expectations related to provider skills were frequently expressed, not least regarding the use of clear language devoid of medical jargon. Respondents also point to the fact that there are sometimes topics, which need to be brought into the daylight that may be difficult to talk about. Providers have to be ready to ask “the uncomfortable questions” and have the ability to create a climate of safety and trust. The users expressed a desire for support in considering the pros and cons that various treatment alternatives may entail and to assess the implications in relation to personal needs, aspirations, values, fears, preferences, and activities. Furthermore, they point to different aspects of personal competence derived from their life and illness experience, including coping strategies, self-knowledge, and self-treatment, as potentially being of great value when developing action plans.

[The provider] could see the person and ask “what strengths does this person have to feel better and to manage in life”. Then they might get support in their treatment and at the same time be reminded to note his strengths and goals in lives. (3)

### Decision talk

In decision talk, Elwyn et al. (2013) suggests that the user is guided in defining what matters most to him or her. The provider should offer time for consideration and opportunities for further discussion regarding concerns and worries if needed. They should also offer to make recommendations if the person so wishes. Similar to this central notion of Elwyn's model, respondents expressed how being allowed time to ponder and deliberate during, as well as in between encounters, is a prerequisite for being involved in a real sense.

Respondents expressed the importance they attached to providers actively requesting information relating to the variety of life domains that the Elwyn model points to, including personal needs, aspirations, values, and activities. From their experience of mental illness however, such inquiry is rarely as thorough as they would wish. Respondents noted how sensitive matters, which might be laden with feelings of fear or shame, are often kept hidden and how providers need to support these coming to light because they might hold crucial clues as to which issues need to be focused upon.

Sometimes I get tongue-tied, don′t know what to say, it′s hard to talk about myself …. Easier if they ask leading questions as to what they need to know … (16).

The respondents in the current study are experienced users, with a long experience of living with their illness, and many noted a desire for their life or experiential knowledge to be confirmed as valuable in the decision-making process. Many noted how the self-help strategies that they had developed might be very relevant to consider when choosing among options because increased self-reliance naturally may allow for a decrease of intensity of system services.

When moving toward a decision, the respondents frequently expressed a wish for the process to be less hurried. They describe how they experience providers working under time pressure and how they sometimes find it difficult to keep pace with them.

You also need time … And the opportunity to come several times. … To go home and think about different options in order to make a decision. (8)

Elwyn et al. (2013) suggests that offering a review of what has been discussed may be helpful in arriving at closure, which is also expressed by the respondents in this study.

Respondents described how discussions in provider encounters often are both diverse and comprehensive and they expressed a wish for written summaries after provider contacts for reducing risks of misunderstandings.

### Follow-up

In the current data, numerous references are made to the need for and possibility of further contacting the provider after a decision has been made. However, many of the ideas regarding how support may be provided at this stage of the process fall outside the scope of Elwyn's model. A fifth phase in the model of SDM in mental health services is therefore suggested as a follow-up phase. For the most part, the decision-making processes to which respondents referred, related to elements and components of care and treatment procedures which are multifaceted, interactive, and long-term and which thereby call for continuous evaluating, adapting, and reevaluating. The importance of having a planned return visit for review and follow-up was therefore emphasized, as was the point of being able to follow one's progress, to know how long a decision will remain in effect, and the possibilities for reviewing or revising a decision.

But it gives you a certain sense of security, if I can get in touch with the doctor … sometimes I don′t remember what we decided about raising or lowering (the dose) …. (10)

Many respondents described how their questions often arise hours, days, or weeks after the latest provider encounter. They noted how sometimes after encounters they feel overloaded with information which might be both detailed and diverse. In addition, some expressed experiencing how the emotional intensity of matters dealt with during encounters may inhibit cognitive functions such as information processing and memory. Discomfort with interacting with authority was also described as another stressor, which might sometimes lead to mental blockage Being offered follow-up contacts by e-mail or telephone if questions should arise was expressed as desirable and could reduce stress related to the decision.

Yes, it would probably be good if you could have an e-mail contact between meetings, … or a phone call. So that you feel that the doctor can follow up on things. (10)

## Discussion

Implementing and promoting SDM in mental health services can support the development of user-centered care and recovery (Matthias et al., 2012; Slade et al., 2012). The results from this study illustrate that the model Elwyn et al. (2013) has created for SDM in somatic care, in general, fits well for mental health services, both in terms of process and content. However, the results suggest two additional steps to the model important for SDM in mental health services. These additions consisted of a *Preparation* phase, which emphasized the importance of developing and describing issues related to provider tasks as well as to user tasks, and a *Follow-up* phase concerning needs and wishes for further contacts with the provider after a decision has been made. The respondents pointed out the importance of the need for support during all phases and the potential benefits of involving others in the process. The advantages of being accompanied by someone who knows you well can have a counterbalancing impact on the power imbalance that often occurs in the meeting with staff. This suggested extension of the model can be understood as an adaptation to the psychosocial context and the specific needs of users with mental illnesses. In medically oriented decision-making processes, the focus of the decision is often clearly established based on the diagnostic circumstances, and the exchange of information (Loh et al., 2007; O'Connor et al., 2007). In community-based mental health services on the other hand, decisions are often complex and involve a number of life domains (Deegan & Drake, 2006). This suggests that issues related to life context and individual recovery need to be considered from the outset in the decision-making process and the knowledge from this study might be usefully applied in attempts in the development of decision support tools in community-based mental health services.

Elwyn et al. (2013) emphasize deliberation in the SDM process as something that begins as soon as awareness about options develops. The process is supposed to be iterative and recursive, and the intensity increases as options are described and considered. The initial or added *Preparation* phase, suggested from the results in this study, can be understood as a precursor to deliberation. The extension is thus a desire for a decision support tool that contributes to a broader approach to illness and how it should be treated, and a desire for a more equal encounter between caregivers and users. These aspects have been outlined as essential in recovery-oriented mental health services (Onken et al., 2007; Slade et al., 2012) and emerge as crucial to an improved decision-making process.

The second suggested additional phase to Elwyn's model is a distinct *Follow-up* process, which clearly defines the ongoing nature of the decision-making process and includes concrete options for reviewing or reconsidering the current decision (Elwyn et al., 2013). Because mental illness and illness management affect such a range of life domains and because effects of changes often are delayed, chains of causes and effects might be difficult to trace. Consequently, a methodical follow-up procedure is often needed for the sake of trying out, monitoring, and modifying interventions (Hamann et al., 2010; Woltmann, Wilkniss, Teachout, McHugo, & Drake, 2010). The statements, in which respondents specify objectives for further communication, reflect how using mental health services often involves care and treatment procedures, which are both multifaceted and often long term. The challenge of decision-making appears to constitute a dynamic and recurrent element in the lives of many respondents for whom dosage and combinations of medicine as well as strategies for day-to-day management might need to be adjusted continually (Deegan & Drake, 2006; Woltmann et al., 2010).

Finally, the respondents, despite their general agreement with the strategies described in the Elwyn model (Elwyn et al., 2013), point to a number of special characteristics, related specifically to the experience of being a user of mental health services. These include issues related to some of the stigmatizing or stereotyped ideas of individuals experiencing mental illness as lacking insight into their needs (Hansson, Jormfeldt, Svedberg, & Svensson, 2013). The respondents also present the particular cognitive challenges, which they face in terms of both taking in and expressing knowledge related to decisions. The decisions themselves are described as complex, containing both biopsychosocial aspects and interactions, as well as having effects and consequences in the individual's life, which only emerge over time. And finally, the non-linear, individual and relationship-based nature of the recovery process (Deegan & Drake, 2006) is exemplified in many of the responses and may be seen as explaining a further dimension of complexity in decision-making processes related to mental health issues.

### Methodological consideration

The findings of this study are limited because of the small amount of respondents. Users of community-based mental health services are a multifaceted group with various needs that vary over time. These needs are not all reflected in the focus groups, despite the fact that respondents with different diagnoses, ages, and experiences were included. However, even if experiences of mental illness and psychosocial interventions are, in one sense, individual and subjective, there is no reason to believe that the respondents’ experiences in this study differ dramatically from others in similar situations. The citations contribute to a certain transparency of the results and the fact that two of the researchers conducted the analysis independently may contribute to the validity of the results.

## Conclusion and implication

The current study not only contributes to a better understanding of decisional and information needs among users with mental illness that may reduce barriers to participation in decision-making processes but also sheds light on attitudinal, relationship-based, and cognitive factors that may be important to consider in adapting the SDM method for individuals in the mental health system. There are many challenges ahead in implementing SDM in clinical encounters, and new systems will be required to appropriately reward patient-centered practice. The introduction of decision support not only helps to enable users to actively participate but also functions as a tool to make SDM a practical reality in mental health services.

Further research will be needed and is underway, to test the model with various populations of users in mental health services and to further develop concrete methods for implementing a SDM process in community-based mental health services. An additional challenge is to further develop an understanding of how participation through methods such as SDM contribute to a recovery-oriented system, as well as a need to study both clinical and satisfaction-related outcomes connected to the method.
